# Feeding amylolytic and fibrolytic exogenous enzymes in feedlot diets: effects on ruminal parameters, nitrogen balance and microbial diversity of Nellore cattle

**DOI:** 10.1186/s40104-025-01226-5

**Published:** 2025-07-07

**Authors:** Igor Machado Ferreira, Hilario Cuquetto Mantovani, Fiorella Viquez-Umana, Yury Tatiana Granja-Salcedo, Luiz Fernando Costa e Silva, Anne Koontz, Vaughn Holder, James Eugene Pettigrew, Arlan Araújo Rodrigues, Aline Naime Rodrigues, Mateus José Inácio de Abreu, Saulo Teixeira Rodrigues de Almeida, Pedro Marcus Pereira Vidigal, Gustavo Rezende Siqueira, Flávio Dutra de Resende

**Affiliations:** 1 Department of Animal Science, Faculty of Agricultural and Veterinary Sciences, São Paulo State University, Jaboticabal, SP 14884-900 Brazil; 2 Agência Paulista de Tecnologia Dos Agronegócios (APTA), Colina, São Paulo, Brazil; 3 Department of Animal and Dairy Sciences, University of Wisconsin-Madison, Madison, WI USA; 4 Corporación Colombiana de Investigación Agropecuaria (AGROSAVIA), Centro de Investigación El Nus, San Roque, Antioquia 053030 Colombia; 5 Alltech, Maringa, Paraná 87030-405 Brazil; 6 Alltech Inc, Nicholasville, KY 40356 USA; 7 Pettigrew Research Services, Tubac, AZ 85646 USA; 8 Department of Animal Science, Federal University of Paraíba, Areia, Paraíba Brazil; 9 Núcleo de Análise de Biomoléculas (NuBioMol), Center of Biological Sciences, Federal University of Viçosa (UFV), Viçosa, MG Brazil

**Keywords:** Amylolytic enzymes, Beef cattle, Fibrolytic enzymes, Nitrogen metabolism, Rumen microbiome

## Abstract

**Background:**

The environmental impact of feedlot operations is a growing concern, as cattle excrete a significant portion of feed nutrients as waste. Exogenous feed enzymes (EFE) have gained interest for their potential to enhance feed efficiency in ruminants by improving nutrient digestion. However, EFE effects on ruminal parameters have shown inconsistencies, with limited research on nitrogen metabolism and rumen microbiome impacts. Moreover, the synergistic effects of combining different EFEs remain unclear. This study aimed to evaluate the effects of individual and combined EFE products in feedlot diets on ruminal fermentation parameters, nitrogen metabolism, and ruminal microbial communities. Ten rumen-cannulated Nellore steers [543 ± 28.6 kg of body weight (BW)] were distributed in a replicated Latin-square design (5 × 5) in individual pens. Treatments included: control (CON, no EFE supplementation), amylase [AML, 0.5 g/kg of diet dry matter (DM)], xylanase (FBL, 0.9 g/kg DM), half dose combination (HD, 0.25 g of AML + 0.45 g of FBL/kg of DM), and full dose combination (FD, 0.5 g of AML + 0.90 g of FBL/kg of DM). The experimental period lasted 19 d and included total urine and feces collection (d 15 to 18) and rumen fluid sampling (d 19) at 0, 4, 8, 12, and 16 h post-feeding for ammonia, volatile fatty acids (VFA), pH and microbiome analysis.

**Results:**

EFE supplemented animals exhibited lower ruminal ammonia concentrations (*P* = 0.040), and higher acetate proportions (*P* < 0.001) compared to the control group. EFE supplementation resulted in reduced nitrogen (N) excretion in feces (*P* = 0.049) and urine (*P* = 0.036), contributing to improved N retention and efficiency (*P* = 0.045). Additionally, EFE products induced shifts in various microbial taxa at family and genera levels (*P* ≤ 0.10), which may be associated with the changes observed in ruminal fermentation.

**Conclusions:**

Our findings demonstrate that EFE supplementation enhances nitrogen retention, reduces ruminal ammonia, and alters ruminal fermentation profiles and microbial populations in feedlot cattle. While the expected synergism between amylase and xylanase did not significantly impact rumen fermentation parameters, it did induce shifts in the rumen microbiome. These results suggest that EFE supplementation may be a promising strategy for improving nutrient utilization and potentially reducing the environmental impact of feedlot operations.

**Supplementary Information:**

The online version contains supplementary material available at 10.1186/s40104-025-01226-5.

## Background

The environmental impact of feedlot operations is becoming a growing global concern, primarily because feed digestion in beef cattle is incomplete. Cattle retain only a portion of the feed nutrients, with the remainder lost to the environment, mainly as feces and urine [1]. Among the feed additives used in ruminant diets, exogenous feed enzymes (EFE) have gained scientific interest as a strategy to optimize animal feed efficiency by enhancing nutrient digestion [2–4]. EFE may enhance feed utilization by increasing the rate and extent of fiber and starch hydrolysis, digestion, and degradation during pre-ingestive, ruminal, and post-ruminal phases. EFE can catalyze direct hydrolysis of feed substrates, which reduces the viscosity of digestive fluids and helps increase ruminal passage rate, [3, 5, 6]. Additionally, the enzymatic hydrolysis facilitates access of rumen microbes to complex, non-soluble substrates, therefore contributing to modulating the ruminal microbial population [7, 8].

Initial studies on EFEs in ruminant nutrition focused on enhancing the rate and/or extent of fiber degradation through fibrolytic enzymes [4]. Exogenous amylolytic enzymes have also been evaluated to optimize ruminal starch utilization [9, 10], aiming to promote changes in ruminal fermentation by increasing total volatile fatty acid concentration. However, the effect of EFE on ruminal parameters has been inconsistent [11, 12]. Furthermore, few studies have considered the effect of EFE supplementation in feedlot diets on nitrogen metabolism, and most studies evaluating EFE supplementation on rumen populations have been conducted in vitro [7, 13] or using dairy cattle as a research model [14–16]. Thus, to the best of our knowledge, this is the first study to evaluate the effects of combined EFE supplementation on the ruminal microbial community of beef cattle.

Recently, promising results regarding total-tract crude protein digestibility in ruminants were reported when fibrolytic (Fibrozyme^®^) and amylolytic (Amaize^®^) enzymes were added to dairy [2] and beef cattle diets [17]. However, the possible synergism between these enzymes in terms of improving nitrogen metabolism in beef cattle has not been explored. The combination of different EFE may allow the breakdown of cross-linkages between structural polysaccharides of the feed, potentially enhancing nutrient utilization [4, 11]. Thus, we hypothesized that feeding fibrolytic and amylolytic enzymes in feedlot diets would alter nitrogen metabolism and total ruminal VFA concentration through modulation of the ruminal microbiome of Nellore cattle. Specifically, the fibrolytic enzymes would target the breakdown of cellulose and hemicellulose, while amylolytic enzymes would enhance starch degradation. The combination of these enzymes could synergistically increase nutrient utilization by breaking down complex polysaccharides and cross-linkages between structural carbohydrates, thereby improving overall feed efficiency. Additionally, we hypothesized that feeding both fibrolytic and amylolytic enzymes would have synergistic effects compared to feeding individual enzymes, due to their complementary actions on different substrates within the feed. The objective of this study was to evaluate the effect of both fibrolytic and amylolytic enzymes added to the feedlot diet on ruminal parameters, microbial diversity, and nitrogen balance of Nellore cattle.

## Materials and methods

### Treatments and experimental design

The study was conducted at the Experimental Feedlot Cattle facilities of the Agência Paulista de Tecnologia dos Agronegócios, Alta Mogiana regional pole, Colina São Paulo, Brazil. Ten rumen-cannulated Nellore steers (543 kg ± 28.6 BW) were distributed in a replicated (5 × 5) Latin-square design. Initially, the animals were identified, treated for internal and external parasites (Dectomax^®^, Zoetis Brasil; Topline^®^, Boehringer Ingelheim, SP, Brazil), housed in individual stalls (2 m × 5 m) with concrete flooring, feed bunks, and free access to water. The experiment had five 19-day periods including 14 days of treatment adaptation [18] and the last 5 d for data collection and sampling. Nitrogen balance was assessed through total fecal and urinary sampling carried out over three consecutive days (d 15 to 18), followed by one day for rumen content collection (d 19; VFA, ruminal ammonia (N-NH_3_), pH, and microbiome).

The animals were assigned to the following treatments: (1) control (CON), basal diet without exogenous enzymes; (2) amylolytic enzyme product added at 0.5 g/kg of diet dry matter (DM; AML, Amaize^®^, Alltech Inc., Nicholasville, KY, USA); (3) fibrolytic enzyme product added at 0.9 g/kg of diet DM (FBL, Fibrozyme^®^, Alltech Inc.); (4) both amylolytic and fibrolytic enzymes added at half the dose of individual products (Half dose; HD); and (5) both amylolytic and fibrolytic enzymes added at the same dose of individual products (Full dose, FD). According to the manufacturer, Fibrozyme^®^ is an extract from *Trichoderma longibrachiatum* fermentation (a dry mixture of inactive yeast, dry brewery yeast, and yucca extract) with a minimum of 100 IU (International unit) of xylanase activity per gram of product. Amaize^®^ consists of an *Aspergillus oryzae* culture extract with a minimum α-amylase activity of 600 FAU (Fungal amylase unit)/g of product. One FAU is defined as the amount of enzyme that degrades 1 g of dextrinized soluble starch per hour at 30 °C and pH of 4.8 [19].

### Feed management

The steers were fed daily (8:00 am) ad libitum a total mixed ration (TMR) consisting of 18.5% of whole corn silage as a roughage source, 41.3% of dry ground corn, 23.6% citrus pulp, 5.60% soybean meal, 7.93% cottonseed and 3.13% of a feedlot premix (Table 1; DM-basis). Before starting the experimental diets, the steers were adapted to the diet for 21 d without EFE supplementation. The concentrate portion and enzyme products (available in a dry powder form) were weighed individually for each animal, and then manually mixed with whole corn silage at the time of feeding. Thus, the enzyme dose was adjusted according to the daily DMI. Feed bunks were evaluated each day to quantify refusals and adjust daily feed allowance to a maximum of 5 % refusals.

**Table 1:** Feed ingredients proportion and chemical composition of the diets

Ingredient, % DM	Acclimation diet	Experimental diet
Whole corn silage	40.7	18.5
Ground corn	23.3	41.3
Citrus pulp	18.6	23.6
Soybean meal	8.74	5.60
Cottonseed	5.61	7.93
Feedlot premix ^a^	3.13	3.13
**Nutritional profile, % DM**		
Dry matter	63.0	65.8
Organic matter	93.9	93.9
Crude protein	15.1	14.1
Neutral detergent fiber	32.7	26.4
Acid detergent fiber	14.4	13.3
Ether extract	3.53	3.82
Non-fiber carbohydrate	47.9	48.5
Starch	31.2	35.6
Total digestible nutrients	73.3	75.8
ME, Mcal/kg ^c^	2.65	2.88
NEm, Mcal/kg ^d^	1.85	2.05
NEg, Mcal/kg ^d^	1.22	1.29

^a^Content per kilogram of product: 116.7 g of Ca, 19.4 g of P; 38.9 g of Na, 29.2 g of S, 1,167 mg of Zn, 9,722 mg of Mg; 386 mg of Cu, 463 mg of F, 778 mg of Mn, 11.4 mg of Co, 19.4 mg of I, 9.4 mg of Se, 808 mg of monensin sodium, and 323 mg of urea. Manufactured by Alltech^®^, Parana, Brazil

^b^Total digestible nutrients (TDN) were estimated using the individual composition of feedstuff according to Valadares Filho et al. [74]

^c^Metabolizable energy (ME) = TDN (g/kg DM) × 4.4 × 0.82 [75]

^d^The net energy for maintenance (NEm) and gain (NEg) were estimated with the equations proposed by NASEM (2016; empirical solution type) with the addition of ionophore and using the TDN values

### Evaluations

**Fig. 1:**
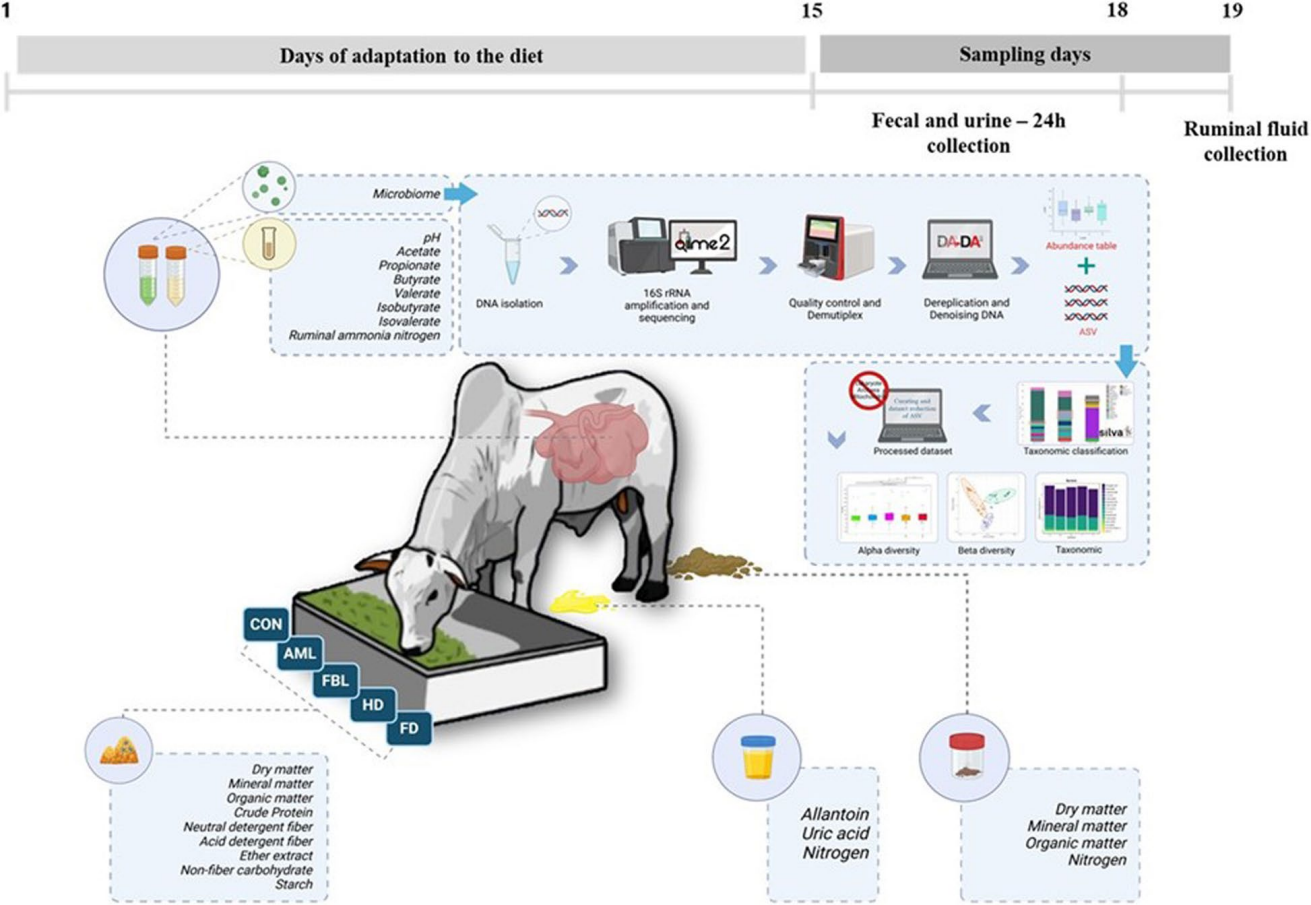
Experimental workflow. Created in https://BioRender.com

All collections were made according to the experiment workflow (Fig. 1) following the respective methodology described below.

#### Ruminal fermentation parameters

Ruminal contents were collected at time 0 (before morning feeding) and 4, 8, 12, and 16 h post-feeding from each cannulated steer on day 19 from the dorsal, central, and ventral regions of the rumen to form a composite sample, which was immediately filtered through two layers of cheesecloth. Liquor samples were placed into 50-mL falcon tubes and were immediately used for ruminal pH measurement using a pH meter (DM-22, Digimed, Sao Paulo, Brazil). Two aliquots from each time point were then placed into a 15-mL conical centrifuge tube, preserved with 1 mL of H_2_SO_4_ (sulfuric acid), and stored at −20 °C until analyzed for ruminal ammonia nitrogen (NH_3_-N) [20]. Another 15 mL was collected for analysis of the molar proportion of volatile fatty acids (VFA; acetate, propionate, butyrate, valerate, isobutyrate, and isovalerate) and stored at −20 °C. A microcentrifuge vial was thawed and processed following standard laboratory procedures. Briefly, a 1.6-mL of ruminal fluid was centrifuged for 30 min at 15,000 × *g* at 4 °C. The supernatant (0.4 mL) was transferred to a chromatography flask and 0.2 mL of a 3:1 solution of metaphosphoric acid (250 mL/L) and formic acid (908–1,000 mL/L) was added. The internal standard was added to each vial (0.1 mL of 100 mmol/L 2-ethyl-butyric acid). Acid separation and analyses were performed using the gas chromatograph (Agilent 7890) equipped with flame ionization detection (7683B) and 25-m long fused silica capillary column (320 µm internal diameter, J & W 19091 F-112, Agilent Technologies, Santa Clara, CA, USA), following the method described by Erwin et al. [21].

#### Microbial efficiency, microbial protein synthesis, and nitrogen use

Total feces and urine collection were performed over 24 h on d 15 to 18. Urine output was collected using collecting funnels attached to animals and coupled with flexible hoses into 20-L containers with 500 mL of 20 g/100 mL sulfuric acid. At the end of the first collection day, the total urine volume was measured using a 2-L measuring cylinder, placed in a 20-L bucket, and mixed manually. Then, a 50 mL aliquot was collected from the total urine volume and diluted (1:4 ratio, vol of urine/vol H_2_SO_4_) into a sulfuric acid solution at 0.018 mol/L*,* placed in a plastic bottle, and stored at −20 °C. The previously added acid solution was excluded from all calculations, so the volume measurements and calculations of total urine volume and urine subsample comprised only urine. Urine and feces samples were grouped to obtain a composite sample based on the daily volume.

Allantoin concentrations in urine were assessed by a colorimetric method according to Chen and Gomes [22], with absorbances measured using a microplate reader (Biochrom Asys UVM 340, Biochrom Asys). Uric acid concentration in urine was analyzed using a commercial kit (Uric Acid Stable Liquid, catalog no. K-052, Bioclin). The daily excretion of allantoin and uric acid is used to estimate microbial protein production in the rumen and was estimated by multiplying the urine concentrations by the average urinary volume. Excretion of the purine derivatives in urine was calculated by the sum of the allantoin and uric acid excretions (mmol/d). Absorbed purines were estimated by derivatives of total purines using the formula: Pa (mmol/d) = [PDe − (0.301 × BW^0.75^)]/0.80, where PDe (mmol/d) is purine derivatives excreted (uric acid and allantoin), BW is body weight, 0.301 × BW^0.75^ represents excretion of endogenous of PDe and 0.80 is the recovery of Pa [23].

Microbial nitrogen synthesis was calculated by absorbed purines using the formula: Nmic (g N/d) = [(70 × Pa)/(0.93 × 1,000 × 0.137)], where 70 represents the concentration of N in purines (mg/mmol), 0.93 is the digestibility of purines and 0.137 is the average ratio of RNA-N to total-N for rumen bacteria [23]. The efficiency of microbial synthesis in the rumen [g microbial N/kg of digestible OM intake (DOMI)] was calculated by dividing the production of ruminal microbial N (g microbial N/d) by the DOMI (kg/d). The CP/DOMI ratio was calculated based on the intake of digestible OM and CP (g/kg).

Nitrogen balance was obtained by subtracting the total excreted N (feces and urine) from total N intake, representing the total N that was effectively retained by the animal. For this purpose, diet components, fecal, and urine samples were analyzed for N (920.87) and ash (942.05) according to AOAC method [24]. Organic matter was calculated as organic matter = 100 – ash. To determine the fecal N excretion (FNE), the fecal production was multiplied by the total N concentration in the feces. Urinary N excretion was calculated by multiplying the average of urinary volume by urinary N concentration. The apparent efficiency of N utilization (ENU) by the animal was calculated by dividing the nitrogen balance/nitrogen intake.

#### Ruminal bacteria and archaea diversity

Ruminal content (solid + liquid) samples of approximately 50 g per animal were collected from the dorsal, central, and ventral regions of the rumen through the ruminal cannula. Collections were made 4 h after steers were fed the diet supplemented with EFE on d 19 and were immediately stored at −80 °C until further analysis. The samples were processed to obtain a microbial pellet [25], with phosphate saline buffer (pH 7.4). For DNA extraction, 250 mg of the microbial pellet was used. A Quick-DNA™ Fecal/Soil Microbe Miniprep kit according to the manufacturer’s instructions (Zymo Research Corporation, CA, United States), and the bead beating method for cell lysis (FastPrep-24, MP, Biomedicals, Illkirch, France) were used. The DNA concentrations were assessed using a spectrophotometer (NanoDrop ND-1000 Spectrophotometer, Thermo Fisher Scientific, Waltham, MA, United States) and a fluorometer (QubitR 3.0, kit Qubit RdsDNA Broad Range Assay Kit, Life Technologies, Carlsbad, CA, USA). DNA purity was confirmed spectrophotometrically by absorbance ratios at 260/230 and 260/280 nm and DNA integrity (no evidence of degradation) was determined by agarose gel electrophoresis using a 0.8% (w/v) gel and stained with SYBR Safe DNA Gel Stains (Thermo Fisher Scientific, Waltham, MA, USA).

Libraries were prepared by PCR amplification of the V3/V4 regions of the 16S ribosomal RNA gene (16S rRNA) using barcoded 16S Illumina primers [26]. Each sample was amplified in duplicates, and each PCR reaction mixture (20 µL final volume) contained 20 ng of metagenomic DNA, 10 µmol/L of each forward and reverse primers, 1.25 mmol/L of magnesium chloride, 200 µmol/L of dNTP mix (Invitrogen, Carlsbad, CA, United States), 1.0 U platinum Taq DNA polymerase high fidelity (Invitrogen, Carlsbad, CA, USA), PCR buffer (1X), and milli-Q water. Reactions were conducted at 95 °C for 3 min to denature the DNA, with amplification proceeding for 30 cycles at 95 °C for 30 s, 53.8 °C for 30 s, and 72 °C for 45 s; a final extension at 72 °C for 10 min was added to ensure complete amplification. The fragment length of PCR products was verified by agarose gel (1%) electrophoresis, using a 1 kb plus DNA ladder (1 kb plus DNA ladder, Invitrogen, Carlsbad, CA, USA) for amplicon size estimation. PCR fragments were purified using the Zymoclean™ Gel DNA Recovery kit following the manufacturer’s instructions, and composite samples for sequencing were created by combining equimolar ratios of amplicons from the duplicate samples. Sequencing was performed using the Illumina MiSeq® platform with MiSeq Reagent v2 (2 × 250 pb; Illumina^®^, USA) kit.

#### Sequencing data clean-up

Sequence data were demultiplexed and quality-filtered using the q2-demux plugin followed by denoising with DADA2 [27]. The samples were rarefied to 17,405 sequences per sample, and the q2-diversity plugin was used to estimate the diversity metrics as alpha-diversity [28] and beta-diversity [29]. All amplicon sequence variants (ASVs) were aligned with mafft [30] and used to construct a phylogeny with fasttree [31]. The ASVs were assembled by the available reference method in the Quantitative Insights into Microbial Ecology (QIIME) software package version 2. Taxonomy was assigned to ASVs using the q2-feature-classifier classify-consensus-search taxonomy classifier against the Silva 128 database with 97% ASVs reference sequences [32].

### Statistical analysis

The effects of enzyme supplementation were performed using contrasts: (1) CON vs. EFEs (2) AML vs. FD, (3) FBL vs. FD, and (4) HD vs. FD. Except for ruminal microbial diversity, all data were analyzed by fitting mixed models [33], using the MIXED procedure of SAS (SAS Institute, Cary, NC, USA). First, the mathematical assumptions of data normality (Shapiro–Wilk test) and homogeneity of variance (Bartlett test) were tested. The data were considered normally distributed (Shapiro–Wilk test, W ≥ 0.80) using the “Univariate” procedure of SAS. The experimental model considered “treatment” as a fixed effect and, “period”, “animal” and “square” as random effects. Variables measured in the same experimental unit over time (rumen pH, NH_3_-N, and VFA) were analyzed as repeated measurements using the “repeated” procedure of SAS. The symmetry of the covariance structure of the repeated measure analyses was chosen based on the lowest BIC (Bayesian Information criterion). The Kenward-Rogers approach approximation was used to adjust the denominator degrees of freedom for the test of fixed effects.

Richness estimators, diversity index, and ruminal microbial relative abundance of bacteria and archaea data were compared between CON vs. EFE using a Kruskal-Wallis test, while the paired Wilcoxon Rank Sum Test was applied to compare (2) AML vs. FD, (3) FBL vs. FD, and (4) HD vs. FD. A permutation test of multivariate homogeneity of group dispersions based on Bray-Curtis as a similarity index was applied using the *vegan* package in R. Spearman’s rank correlations were used to investigate the relationship between important microbial ASVs associated with ruminal fermentation parameters. A correlation plot was performed using the corrplot library. All rumen microbiome analyses were performed using R Software version 4.3.3 [34].

Statistical differences were declared at *P* ≤ 0.05 and trends toward significance were considered when 0.05 < *P* ≤ 0.10 for all variables.

## Results

### Ruminal fermentation parameters

No differences were observed among treatments for ruminal pH and total VFA (*P* > 0.177; Table 2). Additionally, there was no interaction between Treat × Time on ruminal fermentation parameters (*P* > 0.221). Animals fed EFE had 10.2% lower ruminal N-NH_3_ concentration (*P* = 0.040) values and lower molar proportion of isovalerate (*P* = 0.083) than CON steers. However, a higher ruminal proportion of acetate (*P* < 0.001), valerate (*P* < 0.001), and Acetate:Propionate ratio (*P* = 0.008) was observed in steers fed EFE. Steers fed AML tended to have a lower molar proportion of propionate (*P* = 0.081) and butyrate (*P* = 0.077) but showed a higher Acetate:Propionate ratio (*P* = 0.010) compared to animals supplemented with both enzymes in FD. A tendency for an increase in ruminal N-NH_3_ concentration was observed in steers fed FBL (*P* = 0.082), while they presented a lower valerate than steers fed FD (*P* = 0.015). Therefore, animals in the FD treatment had a higher proportion of propionate in the rumen (*P* = 0.034) than those fed HD of both enzymes.

**Table 2:** Effects of EFE on ruminal fermentation parameters in cannulated Nellore finishing steers (*n* = 10)

Items	Treatments^1^					SEM	*P-*value^2^					
	**CON**	**AML**	**FBL**	**HD**	**FD**		**C1**	**C2**	**C3**	**C4**	**Time**	**Treat × Time**
pH ruminal	5.83	5.81	5.85	5.77	5.79	0.055	0.445	0.696	0.177	0.613	< 0.001	0.983
N-NH_3_, mg/dL	9.23	7.89	8.79	8.68	7.78	0.739	0.040	0.846	0.082	0.120	< 0.001	0.507
Total VFA, mmol/L	49.2	45.5	48.0	49.8	48.4	2.54	0.469	0.221	0.853	0.558	< 0.001	0.677
VFA profile, mol/100 mol												
Acetate	61.3	62.9	62.6	62.4	62.2	1.19	< 0.001	0.143	0.447	0.699	< 0.001	0.991
Propionate	22.3	21.3	21.5	21.1	22.4	1.59	0.118	0.081	0.158	0.034	0.028	0.999
Isobutyrate	0.598	0.594	0.612	0.565	0.589	0.062	0.681	0.919	0.526	0.509	< 0.001	0.221
Butyrate	12.3	11.8	12.4	12.5	12.4	0.411	0.940	0.077	0.935	0.598	< 0.001	0.998
Isovalerate	1.84	1.76	1.67	1.79	1.70	0.138	0.083	0.485	0.666	0.284	< 0.001	0.998
Valerate	1.29	1.45	1.37	1.49	1.47	0.174	< 0.001	0.736	0.015	0.547	< 0.001	0.999
Acetate to propionate ratio	2.94	3.25	3.17	3.07	3.08	0.256	0.008	0.010	0.534	0.881	0.003	0.996

^1^Control (CON) = no feed additives; Amylase (AML) = amylolytic enzyme (Amaize, Alltech) added at 0.5 g/kg diet DM; Xylanase (FBL) = fibrolytic enzyme (Fibrozyme, Alltech) added at 0.9 g/kg diet DM; Half dose of Amylase and Xylanase (HD) = amylolytic enzyme added at 0.25 g/kg diet DM and fibrolytic enzyme (Fibrozyme, Alltech) added at 0.45 g/kg diet DM; and Full dose of Amylase and Xylanase (FD) = amylolytic enzyme added at 0.5 g/kg diet DM and fibrolytic enzyme added at 0.90 g/kg diet DM

^2^Contrast: C1 (CON vs. EFE), C2 (AML vs. FD), C3 (FBL vs. FD), and C4 (HD vs. FD)

*SEM *Standard error of the mean

### Nitrogen balance

No difference was observed between treatments for digestible OM intake (DOMI) and total N intake (*P* > 0.324; Table 3). Animals supplemented with AML had lower CP/DOM values (*P* = 0.051) and tended to have lower fecal N excretion (FNE, *P* = 0.059) compared to animals receiving both enzymes in FD. Animals fed EFE excreted lower amounts of nitrogen in both feces (*P* = 0.049; g of N/d) and urine (*P* = 0.036, g of N/d) than those in the CON group. Additionally, UNE expressed as % of N ingested was lower in animals that received EFE in the diets. Thus, animals fed EFE had higher N retention and ENU values (*P* = 0.045) than animals in the CON group. No differences were observed between FBL vs. FD and HD vs. FD in any of the nitrogen balance variables (*P* > 0.218).

**Table 3:** Effect of EFE on nitrogen balance in cannulated Nellore finishing steers (*n* = 10)

Items	Treatments^1^					SEM	*P-*value^2^			
	**CON**	**AML**	**FBL**	**HD**	**FD**		**C1**	**C2**	**C3**	**C4**
DOMI, kg/d	7.92	7.83	7.71	7.97	7.80	0.329	0.619	0.901	0.704	0.497
CP/DOMI, g/kg	174	166	173	172	173	6.55	0.400	0.035	0.919	0.984
N utilization										
Total N intake, g/d	219	210	212	220	216	12.1	0.324	0.416	0.613	0.531
UNE, g of N/d	99.4	92.9	81.1	91.5	84.6	8.24	0.036	0.174	0.558	0.257
UNE, % N ingested	46.2	44.9	38.3	42.1	39.6	3.48	0.072	0.134	0.694	0.471
FNE, g of N/d	87.3	77.0	79.9	82.9	84.6	6.24	0.054	0.059	0.233	0.662
FNE, % N ingested	40.1	37.3	37.7	38.0	39.8	3.84	0.155	0.149	0.218	0.303
N retention, g of N/d	40.7	44.8	50.8	51.5	50.6	6.32	0.036	0.251	0.960	0.900
ENU, g/g	0.187	0.212	0.241	0.234	0.216	0.020	0.045	0.870	0.282	0.425
Microbial efficiency										
MN, g of microbial nitrogen/d	165	199	164	184	185	18.6	0.334	0.561	0.359	0.959
Emic, g of microbial nitrogen/kg DOMI	20.9	25.4	21.0	23.2	24.1	2.34	0.262	0.646	0.285	0.776

^1^Control (CON) = no feed additives; Amylase (AML) = amylolytic enzyme (Amaize, Alltech) added at 0.5 g/kg diet DM; Xylanase (FBL) = fibrolytic enzyme (Fibrozyme, Alltech) added at 0.9 g/kg diet DM; Half dose of Amylase and Xylanase (HD) = amylolytic enzyme added at 0.25 g/kg diet DM and fibrolytic enzyme (Fibrozyme, Alltech) added at 0.45 g/kg diet DM; and Full dose of Amylase and Xylanase (FD) = amylolytic enzyme added at 0.5 g/kg diet DM and fibrolytic enzyme added at 0.90 g/kg diet DM

^2^Contrast: C1 (CON vs. EFE), C2 (AML vs. FD), C3 (FBL vs. FD), and C4 (HD vs. FD)

*CP/DOMI *Ccrude protein/digestible organic matter intake ratio, *DOMI *Digestible organic matter intake, *Emic *Efficiency of microbial nitrogen synthesis, *ENU *Apparent efficiency of nitrogen utilization in the animals’ body, *FNE *Feces nitrogen excretion, *MN *Microbial nitrogen synthesis, *UNE *Urine nitrogen excretion, *SEM *Standard error of the mean

### Rumen microbial diversity

A total of 5,075,284 raw reads were obtained from 50 samples by sequencing. After trimming, the average number of sequences was 89,248 per sample, of which 66.9% were non-chimeric. The supplementation of EFE did not affect alpha diversity and richness indices (*P* > 0.10; Table S1). In the same way, no difference between treatments in ruminal microbial population beta-diversity Bray-Curtis values was observed (*P* > 0.10; Fig. S1).

**Fig. 2:**
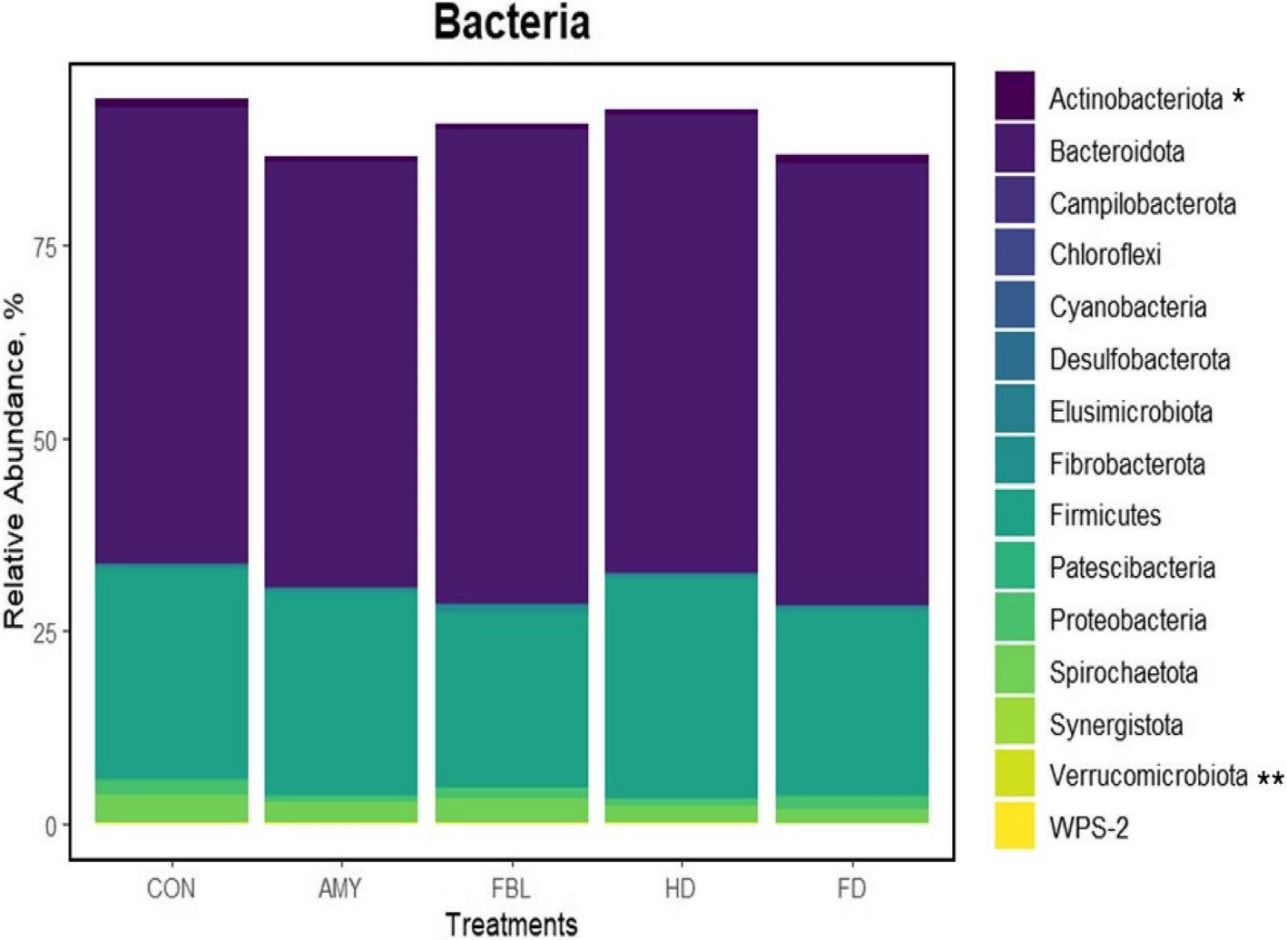
Effects of EFE on the relative abundance of ruminal bacteria at phylum level of cannulated Nellore finishing steers (*n* = 10). * = C3 (FBL vs. FD) = *P* = 0.047; ** = C2 (AML vs. FD) = *P* = 0.066 and C4 (HD vs. FD) = *P* = 0.093

A total of 18 phyla were identified, including two from the Archaea domain (Euryarchaeota and Thermoplasmatota) and 16 from bacteria. The Verrumicrobiota phylum tended to have a lower relative ruminal abundance in animals fed FD (*P* = 0.066; Fig. 2) than in animals fed AML. FD also tended to reduce the ruminal abundance of the Verrucomicrobiota phylum compared to animals on the HD treatment (*P* = 0.093). Additionally, animals that received FBL had a lower relative abundance of Actinobacteriota than those fed FD (*P* = 0.047).

A total of 110 families and 222 genera were identified, and the relative abundance of 14 families was influenced by treatments (Table 4). Within the Actinobacteriota phylum, the Atopobiaceae and Bifidobacteriaceae families tended to have higher abundance in the rumen of FD group (*P* < 0.10) compared to FBL, and the Bifidobacteriaceae family was more abundant in the rumen of animals fed the CON diet (*P* = 0.007) than EFE. In contrast, within Bacteroidota, the Bacteroidales UCG-001, Muribaculaceae, and Bacteroidales uncultured families tended to have lower abundance in animals fed the CON diet compared to those fed EFE (*P* < 0.10). The Bacteroidales RF16 group tended to have higher ruminal abundance in animals fed FD compared to the HD treatment (*P* = 0.059). A lower abundance of the family p-2534-18B5 gut group was observed in animals fed FBL compared to FD (*P* = 0.042).

**Table 4:** Differential bacteria family abundance in cannulated Nellore finishing steers (*n* = 10) feeding EFE

Phylum	Family	Treatments^1^					*P-*value^2^			
		**CON**	**AML**	**FBL**	**HD**	**FD**	**C1**	**C2**	**C3**	**C4**
Actinobacteriota	Atopobiaceae	0.859 ± 1.20	0.522 ± 1.40	0.558 ± 0.384	0.621 ± 0.925	0.807 ± 1.18	0.716	0.799	0.059	0.878
	Bifidobacteriaceae	0.035 ± 0.031	0.009 ± 0.010	NI	0.011 ± 0.022	0.025 ± 0.136	0.007	0.139	0.028	0.214
Bacteroidota	Bacteroidales UCG-001	0.181 ± 0.221	0.113 ± 0.216	0.400 ± 0.164	0.134 ± 0.158	0.224 ± 0.331	0.144	0.093	0.203	0.285
	Bacteroidales RF16 group	0.075 ± 0.176	0.110 ± 0.238	0.041 ± 0.123	0.078 ± 0.068	0.264 ± 0.282	0.706	0.575	0.173	0.059
	Muribaculaceae	6.23 ± 3.98	1.45 ± 3.06	4.61 ± 5.36	3.72 ± 4.14	3.87 ± 7.85	0.099	0.059	0.508	0.721
	p-2534-18B5 gut group	0.033 ± 0.088	0.301 ± 0.655	0.001 ± 0.022	0.009 ± 0.055	0.032 ± 0.422	0.038	0.241	0.043	0.779
	Bacteroidales uncultured	0.391 ± 0.143	0.265 ± 0.173	0.472 ± 0.135	0.420 ± 0.287	0.384 ± 0.221	0.058	0.074	0.333	0.241
Firmicutes	Bacillaceae	0.006 ± 0.012	0.010 ± 0.014	0.002 ± 0.011	0.012 ± 0.034	0.006 ± 0.010	0.641	0.889	0.414	0.074
	Clostridiaceae	0.026 ± 0.072	0.003 ± 0.013	0.014 ± 0.021	0.032 ± 0.025	0.007 ± 0.010	0.008	0.767	0.327	0.021
	Mycoplasmataceae	0.021 ± 0.041	0.029 ± 0.083	0.033 ± 0.144	0.016 ± 0.032	0.021 ± 0.053	0.868	0.799	0.959	0.091
	Ruminococcaceae	2.12 ± 1.25	1.48 ± 1.31	1.52 ± 1.37	2.93 ± 2.45	1.29 ± 0.609	0.188	0.333	0.799	0.009
	UCG-010	0.163 ± 0.116	0.287 ± 0.190	0.186 ± 0.194	0.127 ± 0.180	0.144 ± 0.056	0.386	0.037	0.203	0.721
Proteobacteria	Rickettsiales uncultured	0.021 ± 0.061	0.033 ± 0.076	0.024 ± 0.056	0.046 ± 0.175	0.010 ± 0.016	0.364	0.021	0.114	0.038
Verrucomicrobiota	WCHB1-41	0.032 ± 0.036	0.023 ± 0.052	0.012 ± 0.079	0.018 ± 0.017	0.007 ± 0.012	0.088	0.038	0.074	0.093

^1^Control (CON) = no feed additives; Amylase (AML) = amylolytic enzyme (Amaize, Alltech) added at 0.5 g/kg diet DM; Xylanase (FBL) = fibrolytic enzyme (Fibrozyme, Alltech) added at 0.9 g/kg diet DM; Half dose of Amylase and Xylanase (HD) = amylolytic enzyme added at 0.25 g/kg diet DM and fibrolitic enzyme (Fibrozyme, Alltech) added at 0.45 g/kg diet DM; and Full dose of Amylase and Xylanase (FD) = amylolytic enzyme added at 0.5 g/kg diet DM and fibrolitic enzyme added at 0.90 g/kg diet DM

^2^ C1 (CON vs. EFE) using the Kruskal-Wallis test. Paired Wilcoxon Rank Sum Test was applied to compare C2 (AML vs. FD), C3 (FBL vs. FD), and C4 (HD vs. FD)

*NI *Not identifield

For the Firmicutes phylum, the FD treatment had lower ruminal abundance of the *Clostridium methylpentosum* group and the Ruminococcaceae family (*P* < 0.01) and tended toward lower relative abundance of Bacillaceae compared to HD (*P* = 0.074), and higher ruminal abundance of Mycoplasmataceae (*P* = 0.093). Additionally, the family UCG-010 was more abundant in the rumen of animals fed AML (*P* = 0.037), compared to FD.

In the Proteobacteria phylum, the Rickettsiales uncultured family (*P* < 0.05) was more abundant in the rumen of animals fed AML and HD than those fed FD. For the Verrucomicrobiota phylum, the family WCHB1-41 had a higher relative abundance in animals fed AML than those fed FD (*P* = 0.038), and FD treatment tended to have lower relative abundance of this family when compared to HD, FBL, and CON (*P* < 0.10).

The FD of both enzymes promoted shifts in 21 genera when compared to the AML treatment. Specifically, the *Prevotellaceae NK3B31 group* and *SP3-e08* were higher in the rumen of FD (*P* < 0.05; Table 5). Conversely, several genera, including *Amnipila,* *Lachnospiraceae* *XPB1014 group, Hydrogenoanaerobacterium, Incertae Sedis, Selenomonas, Anaerovibrio,* *UCG-010,* *Christensenellaceae* *uncultured, Rhodospirillales uncultured, Rickettsiales uncultured, Sphaerochaeta* and *WCHB1-41* were higher in the rumen of AML. In addition, animals fed FD tended to have a higher ruminal abundance of *Bacteroidales UCG-001,* *Muribaculaceae unclassifield, Kandleria,* and *Roseburia*, and tended to have a lower ruminal abundance of *Anaerovorax,* *Ruminococcaceae unclassified,* and *Veillonellaceae UCG-001* compared to those fed AML (*P* < 0.10).

**Table 5:** Differential bacteria genera abundance in cannulated Nellore finishing steers (*n* = 10) feeding EFE

Phylum	Genera	Treatments^1^					*P-*value^2^			
		**CON**	**AML**	**FBL**	**HD**	**FD**	**C1**	**C2**	**C3**	**C4**
Actinobacteriota	*Olsenella*	0.782 ± 1.01	0.398 ± 1.38	0.410 ± 0.311	0.541 ± 0.863	0.675 ± 1.24	0.660	0.878	0.059	0.878
	*Bifidobacterium*	0.030 ± 0.039	0.009 ± 0.012	NI	0.011 ± 0.015	0.022 ± 0.109	0.015	0.139	0.028	0.214
Bacteroidota	*Bacteroidales RF16 group*	0.075 ± 0.176	0.110 ± 0.238	0.041 ± 0.123	0.078 ± 0.068	0.264 ± 0.282	0.706	0.575	0.173	0.059
	*Bacteroidales UCG-001*	0.181 ± 0.221	0.113 ± 0.216	0.400 ± 0.164	0.134 ± 0.158	0.224 ± 0.331	0.144	0.093	0.203	0.285
	*Muribaculaceae unclassified*	6.23 ± 3.98	1.447 ± 3.06	4.61 ± 5.36	3.72 ± 4.14	3.87 ± 7.85	0.099	0.059	0.508	0.721
	*p-2534-18B5 gut group*	0.033 ± 0.088	0.301 ± 0.655	0.001 ± 0.022	0.009 ± 0.055	0.032 ± 0.422	0.038	0.241	0.043	0.779
	*Prevotella*	31.8 ± 10.9	35.6 ± 13.2	27.8 ± 8.94	35.4 ± 15.3	24.7 ± 13.4	0.368	0.241	0.508	0.047
	*Prevotellaceae* *UCG-003*	0.830 ± 0.862	0.508 ± 0.862	1.294 ± 1.33	0.877 ± 0.492	0.950 ± 0.773	0.498	0.333	0.037	0.646
	*Prevotellaceae* *YAB2003 group*	0.201 ± 0.240	0.075 ± 0.077	0.149 ± 0.083	0.097 ± 0.126	0.142 ± 0.117	0.077	0.241	0.508	0.646
	*Prevotellaceae* *NK3B31 group*	0.425 ± 0.879	0.043 ± 0.083	0.353 ± 1.400	0.132 ± 0.623	0.712 ± 3.07	0.069	0.007	0.445	0.203
	*SP3-e08*	0.687 ± 1.41	0.068 ± 0.611	0.082 ± 0.871	0.349 ± 1.12	0.453 ± 1.18	0.612	0.050	0.735	0.779
	*U29-B03*	0.102 ± 0.117	0.055 ± 0.086	0.105 ± 0.149	0.119 ± 0.078	0.037 ± 0.049	0.059	0.203	0.007	0.059
	*Uncultured*	0.391 ± 0.143	0.265 ± 0.173	0.472 ± 0.135	0.420 ± 0.287	0.384 ± 0.221	0.058	0.074	0.333	0.241
Firmicutes	*Mogibacterium*	0.016 ± 0.015	0.006 ± 0.014	0.010 ± 0.010	0.014 ± 0.010	0.013 ± 0.014	0.556	0.515	0.953	0.093
	*Eubacterium brachy group*	0.030 ± 0.041	0.026 ± 0.036	0.020 ± 0.021	0.036 ± 0.039	0.016 ± 0.017	0.402	0.173	0.028	0.114
	*Anaerovorax*	0.084 ± 0.044	0.132 ± 0.083	0.089 ± 0.077	0.064 ± 0.115	0.077 ± 0.079	0.570	0.086	0.203	0.508
	*Amnipila*	NI	0.006 ± 0.008	0.001 ± 0.007	NI	NI	0.326	0.027	0.244	0.892
	*Bacillus*	0.006 ± 0.012	0.010 ± 0.012	0.002 ± 0.011	0.012 ± 0.034	0.006 ± 0.010	0.658	0.889	0.414	0.074
	*Clostridium methylpentosum group*	0.026 ± 0.072	0.003 ± 0.013	0.014 ± 0.021	0.032 ± 0.025	0.007 ± 0.010	0.008	0.767	0.327	0.021
	*Kandleria*	0.006 ± 0.022	0.004 ± 0.017	0.009 ± 0.016	0.010 ± 0.015	0.018 ± 0.011	0.803	0.093	0.208	0.203
	*Roseburia*	0.134 ± 0.172	0.083 ± 0.057	0.109 ± 0.141	0.102 ± 0.216	0.161 ± 0.459	0.592	0.093	0.169	0.241
	*Lachnospiraceae* *XPB1014 group*	NI	0.037 ± 0.053	0.005 ± 0.033	NI	0.014 ± 0.021	0.300	0.038	0.612	0.889
	*Lachnospiraceae* *FE2018 group*	NI	0.016 ± 0.061	0.009 ± 0.065	0.016 ± 0.033	0.013 ± 0.073	0.093	0.859	0.889	0.721
	*Howardella*	0.003 ± 0.010	0.010 ± 0.014	0.010 ± 0.012	NI	0.011 ± 0.005	0.054	0.386	0.047	0.020
	*Mycoplasma*	0.021 ± 0.041	0.029 ± 0.083	0.033 ± 0.144	0.016 ± 0.032	0.021 ± 0.053	0.868	0.799	0.959	0.091
	*Papillibacter*	0.144 ± 0.255	0.110 ± 0.203	0.265 ± 0.267	0.068 ± 0.164	0.081 ± 0.067	0.624	0.214	0.037	0.959
	*Hydrogenoanaerobacterium*	NI	NI	NI	NI	NI	0.229	0.049	0.518	0.516
	*Ruminococcus*	1.52 ± 0.889	1.04 ± 0.766	1.10 ± 1.02	1.43 ± 1.68	0.988 ± 0.595	0.401	0.959	0.959	0.017
	*Ruminococcaceae unclassified*	0.033 ± 0.058	0.049 ± 0.109	0.022 ± 0.036	0.033 ± 0.044	0.021 ± 0.024	0.335	0.051	0.374	0.086
	*Incertae Sedis*	0.023 ± 0.040	0.029 ± 0.032	0.023 ± 0.026	0.007 ± 0.033	0.008 ± 0.018	0.705	0.035	0.110	0.333
	*Selenomonas*	0.418 ± 0.571	0.301 ± 0.242	0.400 ± 0.530	0.198 ± 0.134	0.126 ± 0.149	0.197	0.037	0.074	0.037
	*Veillonellaceae* *UCG-001*	0.292 ± 0.165	0.257 ± 0.281	0.164 ± 0.257	0.246 ± 0.238	0.159 ± 0.117	0.430	0.074	0.139	0.203
	*Anaerovibrio*	0.070 ± 0.146	0.100 ± 0.100	0.105 ± 0.192	0.037 ± 0.094	0.026 ± 0.055	0.294	0.008	0.021	0.241
	*UCG-010*	0.163 ± 0.116	0.287 ± 0.190	0.186 ± 0.194	0.127 ± 0.180	0.144 ± 0.056	0.386	0.037	0.203	0.721
	*Christensenellaceae uncultured*	0.025 ± 0.013	0.023 ± 0.037	0.031 ± 0.043	0.010 ± 0.030	0.013 ± 0.027	0.511	0.028	0.011	0.959
Proteobacteria	*Rhodospirillales uncultured*	0.021 ± 0.061	0.033 ± 0.076	0.024 ± 0.056	0.046 ± 0.175	0.010 ± 0.016	0.364	0.021	0.114	0.038
	*Rickettsiales uncultured*	NI	0.001 ± 0.004	NI	0.003 ± 0.005	NI	0.040	0.042	0.180	0.017
Spirochaetota	*Sphaerochaeta*	0.156 ± 0.317	0.554 ± 0.370	0.199 ± 0.223	0.128 ± 0.301	0.211 ± 0.294	0.023	0.017	0.721	0.878
Verrucomicrobiota	*WCHB1-41*	0.032 ± 0.036	0.023 ± 0.052	0.012 ± 0.079	0.018 ± 0.017	0.007 ± 0.012	0.088	0.038	0.074	0.093

^1^Control (CON) = no feed additives; Amylase (AML) = amylolytic enzyme (Amaize, Alltech) added at 0.5 g/kg diet DM; Xylanase (FBL) = fibrolytic enzyme (Fibrozyme, Alltech) added at 0.9 g/kg diet DM; Half dose of Amylase and Xylanase (HD) = amylolytic enzyme added at 0.25 g/kg diet DM and fibrolitic enzyme (Fibrozyme, Alltech) added at 0.45 g/kg diet DM; and Full dose of Amylase and Xylanase (FD) = amylolytic enzyme added at 0.5 g/kg diet DM and fibrolitic enzyme added at 0.90 g/kg diet DM

^2^ C1 (CON vs. EFE) using the Kruskal-Wallis test. Paired Wilcoxon Rank Sum Test was applied to compare C2 (AML vs. FD), C3 (FBL vs. FD), and C4 (HD vs. FD)

*NI *Not identifiel

The FD of both enzymes resulted in a higher ruminal relative abundance of genera *Bifidobacterium*, *p-2534-18B5 gut group*, and *Howardella* (*P* < 0.05), and tended toward a higher relative abundance of *Olsenella* (*P* = 0.059) compared to FBL. In contrast, animals fed FBL had a higher relative ruminal abundance of *Prevotellaceae UCG-003, U29-B03*, *Eubacterium brachy* group, *Papillibacter*, *Anaerovibrio* (*P* < 0.05), and tended to a lower abundance of *Selenomonas* and *WCHB1-41* (*P* < 0.10) compared to those fed FD.

The FD treatment promoted a higher relative abundance of the genera *Howardella* (*P* < 0.05) and tended to have higher abundance of *Bacteroidales RF16* *group*, and *Mycoplasma* (*P* < 0.10) compared to both enzymes in HD*.* In contrast, the HD treatment resulted in a higher relative abundance of *Prevotella, Clostridium methylpentosum* group, *Ruminococcus, Selenomonas, Rhodospirillales uncultured, Rickettsiales uncultured* (*P* < 0.05), and tended toward higher abundance of *U29-B03, Mogibacterium, Bacillus*, and *Ruminococcaceae unclassified* compared to FD.

**Fig. 3:**
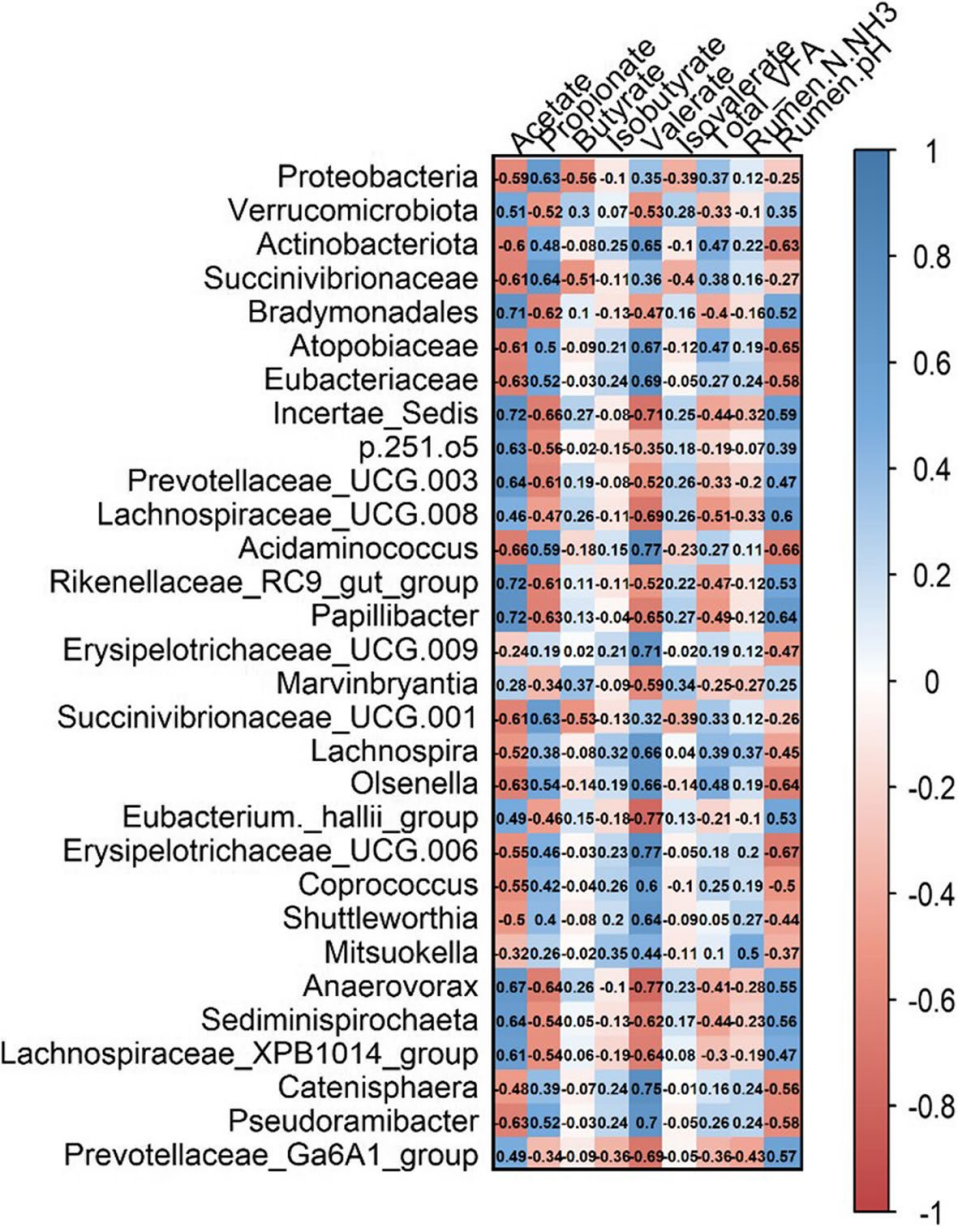
Correlation between rumen fermentation parameters and relative abundances of rumen bacteria in cannulated Nellore finishing steers (*n* = 10)

The ruminal proportion of acetate was positively correlated with *Papillibacter* (0.72), *Incertae Sedis* (0.72), *Prevotellaceae* *UCG-003* (0.64), *Acidaminococcus* (0.66), *Bradymonadales* (0.71), *Rikenellaceae* *RC9* *gut group* (0.72), *Anaerovorax* (0.67), *Sediminispirochaeta* (0.64), and *Lachnospiraceae* *XPB1014* *group* (0.61) relative abundance (Fig. 3). In contrast, the abundance of Proteobacteria phylum (−0.59), Actinobacteriota phylum (−0.60), Succinivibrionaceae (−0.61), *Olsenella* (−0.63), and *Pseudoramibacter* (−0.63) were negatively correlated with ruminal acetate.

The ruminal proportion of propionate showed positive correlations with Proteobacteria phylum (0.63), Succinivibrionaceae (0.64), and *Acidaminococcus* (0.59), and was negatively correlated with *Incertae Sedis* (−0.66), *Anaerovorax* (−0.64), *Papillibacter* (−0.63), *Prevotellaceae* *UCG-003*, *Rikenellaceae RC9* *gut group* (−0.61), and *Bradymonadales* (−0.62). Conversely, the ruminal proportion of butyrate was negatively correlated with the phyla Proteobacteria (−0.56) and Verrucomicrobiota (−0.52), and the Succinivibrionaceae family (−0.51).

The ruminal proportion of valerate was positively correlated with *Coprococcus* (0.60), *Shuttleworthia* (0.64), Actinobacteriota phylum (0.65), *Lachnospira* (0.66), *Olsenella* (0.66), Atopobiaceae (0.67), Eubacteriaceae (0.69), *Pseudoramibacter* (0.70), *Erysipelotrichaceae* *UCG-009* (0.71), *Catenisphaera* (0.75), *Erysipelotrichaceae* *UCG-006* (0.77) and *Acidaminococcus* (0.77). Conversely, negative correlations were observed with *Eubacterium hallii* group (−0.77), *Anaerovorax,* (−0.77), *Incertae Sedis* (−0.71), *Lachnospiraceae* *UCG-008* (−0.69), *Prevotellaceae* *Ga6A1* *group* (−0.69), *Papillibacter* (−0.65), *Lachnospiraceae* *XPB1014 group* (−0.64), and *Sediminispirochaeta* (−0.62).

Finally, rumen pH was associated positively with the relative abundance of *Incertae Sedis* (0.59), *Lachnospiraceae* *UCG-008* (0.60), and *Papillibacter* (0.64), and negatively with *Erysipelotrichaceae* *UCG-006* (−0.67), *Acidaminococcus* (−0.66), Atopobiaceae (−0.65), *Olsenella* (−0.64), Actinobacteriota (−0.63), Eubacteriaceae (−0.58), and *Pseudoramibacter* (−0.58).

The ruminal proportion of isovalerate, isobutyrate, Total VFA, and rumen N-NH_3_ showed weak correlations with the abundance of rumen microorganisms (*r* < 0.50).

## Discussion

We investigated the effect of adding EFE products in feedlot diets on fermentation parameters, nitrogen metabolism, and microbial communities in the rumen of Nellore cattle. We found no differences in ruminal pH and total VFA production. However, our findings reveal that EFE products resulted in lower ruminal N-NH_3_ concentration, shifted VFA profile in the rumen, and reduced nitrogen excretion through feces and urine, consequently leading to greater N retention in the body and improved ENU. Additionally, although we did not observe a significant difference in alpha and beta diversity indices of microbial communities in the rumen, some changes in ruminal fermentation can be associated with shifts in bacterial taxa observed across treatments.

### Effect on ruminal fermentation

The main objective when evaluating EFE in ruminant nutrition is related to the primary effects of the molecule on rumen fermentation. Typically, the increase in animal performance observed when animals are fed EFE has been correlated with increased hydrolytic activity in the rumen and total-tract digestibility of nutrients [3], which might contribute to changes in VFA production and their profiles. Nevertheless, the addition of EFE to feedlot diets has not shown a consistent positive impact on ruminal fermentation [11, 12, 35]. Some factors, like enzyme stability in the rumen [3], enzyme type, and characteristics of the diets being tested [36] have contributed to the inconsistency in the ruminal fermentation of cattle fed EFE. The results of the current study align with the small effects of enzymes on ruminal fermentation reported in the literature [37–40].

Our results related to total VFA production and pH were contrary to our initial hypothesis that the addition of EFE in the diet would impact the ruminal fermentative activity, as demonstrated in in vitro [41] and in vivo studies with dairy [2] and beef cattle on pasture [42] and in feedlots [17]. In the current study, the lack of treatment effect on the DOMI corroborates with the data on the total concentration of VFA. In agreement with our results, Hristov et al. [11] found no effect on the ruminal pH and total VFA production in lactating cows fed both xylanase and α-amylase. Additionally, it is important to highlight that the combined use of EFE (HD or FD treatment) exerted minimal influence on most ruminal parameters (except for propionate and butyrate). This suggests that the increased enzyme dosage may have induced competition with microbial enzyme production, without significantly altering overall ruminal enzymatic activity. Therefore, to maximize the efficacy of EFE supplementation, it is crucial that these exogenous enzymes complement, rather than replace the intrinsic enzymatic capabilities of ruminal microbes.

The addition of EFE promoted changes in the ruminal VFA profile, which may be related to changes in bacterial population (discussed below) due to the hydrolysis of complex carbohydrates by exogenous enzymes and release of different oligosaccharides (malto-, cello-, and xylo-oligosaccharides) that can be used as substrates by multiple species of rumen bacteria. Thus, as postulated by Zilio et al. [2], we expected a synergistic effect between the exogenous enzymes used in the present study because oligosaccharides generated in the rumen can be used by amylolytic and nonamylolytic bacteria in cross-feeding mechanisms [43]. Microbial metabolism of these substrates could lead to changes in ruminal fermentation patterns, including the greater acetate proportion and acetate:propionate ratio observed in the current study.

Considering that acetate is the main product of carbohydrate fermentation by fibrolytic bacteria [44], improved access to the fiber fraction of the diet by rumen microorganisms could potentially drive an increase in the molar proportion of acetate. This is further supported by the enrichment of specific carbohydrate-degrading bacterial genera (discussed below) and a numerical increase of +1.28% in the total tract neutral detergent fiber digestibility coefficient observed in EFE-supplemented animals compared to the control group (unpublished data). It has been reported that exogenous enzymes present synergy with fibrolytic enzymes of rumen microorganisms and promote attachment of rumen microorganisms to plant fiber [5]. Ribeiro et al. [45] reported that an exogenous enzyme induced shifts in rumen microbiota, increased ruminal bacterial diversity (evenness), and decreased protozoa count in lambs.

Animals fed with EFE tended to have a lower isovalerate proportion in the rumen. In contrast, the proportion of isobutyrate was unchanged but the concentration of valerate was higher in animals fed EFE. In addition, the 10.2% lower ruminal N-NH_3_ concentration in animals fed with EFE compared to the control group might indicate that EFE contributed to reducing ruminal proteolysis, altering the degraded protein profile, and preserving the main branched-chain amino acids used to form isoacids in the rumen (leucine, isoleucine, and valine) during proteolytic fermentation [46]. These findings suggest that EFE may have contributed to a greater influx of amino acids into the intestine [47].

Ruminal N-NH_3_ concentration represents a net balance between ammonia production (degraded dietary protein), ammonia uptake by microbes (that varies with carbohydrate availability), ammonia absorption (which varies with ruminal pH), as well as recycling of urea to the rumen (via saliva and through the ruminal wall). In the current study, the rumen degradable protein was similar among diets, ruminal pH did not differ between treatments, and EFE did not significantly impact microbial yield or recycling urea. One possible explanation is that exogenous enzymes may facilitate a more balanced and gradual release of energy (from carbohydrates) and nitrogen (from protein degradation), allowing for more efficient microbial capture of ammonia for growth. Alternatively, the observed lower ruminal N-NH_3_ concentration may be related to slower rates of amino acid deamination in animals fed with EFE. This interpretation is partially supported by a lower relative abundance of members of the Clostridiaceae family and the *Clostridium methylpentosum* group in the EFE treatment. These taxa are known contributors to proteolytic and deamination processes in the rumen [48], and reduction in their populations could decrease ruminal ammonia and impact the profile of isoacids, indicating a shift in nitrogen metabolism toward enhanced nitrogen retention.

Our results are in line with the results of He et al. [49], who evaluated the effect of fibrolytic enzymes in barley-based diets containing wheat-dried distillers grain with solubles in feedlot. Moreover, ruminal protein degradation is an inefficient process due to excessive ammonia production relative to microbial demand [50]. Consequently, excess of ruminal N-NH_3_ is absorbed through the rumen wall and excreted in urine, significantly reducing ENU by the animal [51, 52].

### Effect on nitrogen balance

Beef cattle retain only a portion of the nutrient intake (~ 20%), with the remainder lost mainly in feces and urine [1]. Typically, EFE are added to beef cattle diets to increase nutrient availability, and therefore improve nutrient utilization and feed efficiency [3, 53]. Although few studies report lower nutrient excretion with EFE, our results demonstrated that EFE promoted changes in the nitrogen balance of animals, resulting in lower N excretion in feces and urine.

Animals fed diets treated with EFE excreted an average of 12% less fecal nitrogen compared to the control group, likely due to less excretion of indigestible protein, a consequence of numerically greater crude protein (CP) digestibility of the feed (unpublished data). However, due to the potential reduction in ruminal proteolytic capacity in animals fed EFE, it is possible that EFE may have contributed to greater intestinal digestion activity or improved post-ruminal nutrient absorption [3], possibly resulting in a greater flow of amino acids to the intestine. Supporting this, Nascimento [17] observed that animals fed with α-amylase in a high-grain finishing diet had higher CP digestibility and ENU without a change in ruminal N-NH_3_. Furthermore, Morgavi et al. [54], noted that some exogenous enzymes withstand ruminal fermentation and the abomasal environment, potentially exerting activity in the small intestine. To our knowledge, this study is only the second to investigate the potential influence of EFE on the nitrogen balance of Nellore cattle in a feedlot. Consequently, further studies are needed to better understand the post-ruminal impact of EFE and unveil the mechanisms potentially influencing CP digestibility in feedlot diets treated with EFE.

The nitrogen in feces (undigested food, microbial, and endogenous protein) differs substantially from the N in urine (urea, hippuric acid, allantoin, creatinine, ammonia, and uric acid). Considering the main sources of N excreted via urine, our data regarding the lower concentration of N-NH_3_ in the rumen align with the lowest values ​​of N excretion in urine, since no change was observed in the concentration of purine derivatives used to estimate microbial protein synthesis. Approximately 35%–65% of ammonia is absorbed across the epithelium of the ruminal wall [55], accounting for up to 50% of the total ammonia flow to the liver [56]. Thus, the ammonia that reaches the portal blood is captured and converted to urea in the liver, with some of this urea being recycled (via saliva and across the epithelium of the rumen and other sections of the gastrointestinal tract) or excreted in the urine. In our study, the lower values of N excretion in urine contribute in part to higher ENU and N retention in animals fed with EFE, which was associated with an increase of 2.8 kg of hot carcass weight (unpublished data) compared to animals in the control group. As reported by Alves et al. [52], urinary excretion of urea, synthesized from excess ammonia absorbed from the rumen, is the main factor contributing to the low efficiency of N utilization in Nellore cattle.

Based on a typical 107-day Brazilian feedlot cycle [57], beef cattle fed EFE are estimated to excrete 1.93 kg less nitrogen (fecal and urinary). Focusing solely on nitrous oxide (N_2_O) emissions from excreta and applying emission factors for feces (0.27%) and urine (0.63%) established by Cardoso et al. [58] under tropical conditions, this reduction in nitrogen excretion could lead to a possible 10.7% decrease in N_2_O emissions per feedlot cycle. While these findings suggest that EFE supplementation may improve nitrogen retention and enhance the efficiency of nitrogen utilization (ENU), the extent to which it directly reduces N_2_O emissions remains uncertain and warrants further investigation.

### Effect on ruminal microbial population

To manipulate ruminant efficiency, it is important to understand the role that ruminal microbiota plays in feed utilization by the host [52]. However, few studies have demonstrated the effect of EFE on rumen bacteria and their correlations with host biology.

The lack of difference in beta-diversity and alpha-diversity indices with EFE suggests that the overall rumen microbial community structure remains relatively stable. In line with this, Chung et al. [14] reported that exogenous fibrolytic enzyme supplementation did not alter the population richness of total protozoa, bacteria, and methanogens in the rumen of dairy cows. Similarly, Liu et al. [15] found no impact on rumen bacteria richness and diversity indices when dairy cows were fed a combination of fibrolytic and amylolytic enzymes. Interestingly, both studies also did not detect changes in the final products of fermentation, such as VFA and rumen pH.

However, while overall richness and diversity might not change, compositional shifts in key microbial groups can still have important implications for rumen fermentation and animal performance. In this context, the FD treatment with both enzymes promoted shifts in 21 genera compared to the AML treatment. A lower abundance of Verrucomicrobiota phylum and its genera *WCHB1-41* was observed with the FD treatment. This phylum has been linked to fiber degradation, containing groups of bacteria considered specialized degrading complex polysaccharides, such as *WCHB1-41* [59, 60]. FD also resulted in lower abundance of several fiber-degrading groups from the Firmicutes phylum, such as *Ruminococcaceae unclassified*, *Lachnospiraceae XPB1014 group*, *Incertae Sedis*, *Christensenellaceae uncultured*, *Selenomonas*, *Anaerovibrio*, *UCG-010*, *Hydrogenoanaerobacterium*, *Anaerovorax*, and *Veillonellaceae* *UCG-001* [61, 62], while promoting the enrichment of carbohydrate-degrading bacterial genera, such as *Prevotellaceae NK3B31*, *Bacteroidales UCG-001*, *Muribaculaceae unclassifield*, *Kandleria*, and *Roseburia* [63–65]. This shift is consistent with the greater ruminal acetate proportions observed in animals receiving the FD treatment, and supports possible modification in the fiber fermentation pathways, diverting fermentation to more readily available carbohydrates due to improved access to the fiber fraction of the diet. Additionally, the data suggest that differences among treatments in the abundance of the genera *Lachnospiraceae XPB1014 group*, *Incertae Sedis*, and *Anaerovorax* are associated with the proportions of acetate and propionate in the rumen, including the lower acetate:propionate ratio in FD compared to AML.

In contrast, cattle fed the fibrolytic (FBL) and amylolytic (AML) enzyme treatment had a lower relative abundance of Actinobacteriota phylum compared to FD. This was mainly due to the modulation of the abundance of Atopobiaceae and Bifidobacteriaceae families and their respective genera *Olsenella* and *Bifidobacterium.* Current studies have reported that rumen valerate levels are associated with the *Olsenella* population in ruminants [66, 67]*.* This is consistent with the lower valerate ruminal proportion observed in the FBL group compared to FD, and the association detected between rumen valerate proportion and the Atopobiaceae family and its genus *Olsenella* in our study. *Bifidobacterium* can grow on various carbohydrates, mainly dietary carbohydrates and host-derived glycans [68]. The higher abundance of *Bifidobacterium* with the FD treatment compared to FBL may indicate a higher dietary carbohydrate availability promoted by the combination of enzymes.

The observed differences in microbial populations between the FD and HD of both enzymes highlight how variations in enzyme dosing may shift the balance between carbohydrate and fiber degraders during rumen fermentation. The greater relative abundance observed with FD of the genera *Howardella*, *Bacteroidales RF16* *group*, and *Mycoplasma* is notable. *Howardella* is considered a strong ureolytic genus [69]. The *Bacteroidales RF16* *group* has been related to fibrolytic isozyme activity [70], and *Mycoplasma* was found in microbial consortia during the digestion of low-quality feeds [71]. In contrast, the HD treatment resulted in greater populations of genera from the Firmicutes phylum linked to fiber degradation, such as the *Ruminococcus*, *Ruminococcaceae unclassified*, *Bacillus*, *Mogibacterium*, *Clostridium methylpentosum* group, and *Selenomonas* [72, 73], as well as *Prevotella,* the most abundant genera in the rumen belonging to the Bacteroidetes phylum. *Prevotella* is known as the major pectinolytic bacteria and an active proteolytic taxa in the rumen [48]. Despite the insights from the current study, additional research is needed to establish a causal association between exogenous feed enzymes and fiber digestibility in the rumen.

## Conclusion

This study demonstrated that supplementation with exogenous feed enzymes (EFE) led to lower nitrogen excretion through feces and urine, resulting in greater nitrogen retention by the host. Additionally, EFE supplementation resulted in lower ammonia levels in the rumen and promoted shifts in the ruminal fermentation profile and microbial population. While combining amylase and xylanase did not significantly affect total ruminal fermentation parameters, it did induce shifts in their profile and microbial population. Further studies should further explore the impact of enzyme combinations and dosage rates on ruminal parameters, nitrogen metabolism, and ruminal microbiome populations in a high-starch diet.

## Supplementary Information

Supplementary Material 1: Table S1 Total ASVs, richness and diversity index in cannulated Nellore finishing steers (*n* = 10) feeding EFE. Fig. S1 Principal Coordinate Analysis plot showing the variation between bacterial communities of cannulated Nellore finishing steers (*n* = 10) feeding diets with or without EFE.

## Data Availability

All data generated or analysed during this study are included in this published article and its supplementary information files.

## Abbreviations

AML: Amylase treatment
ASV: Amplicon sequence variants
BW: Body weight
CON: Control treatment
CP: Crude protein
DM: Dry matter
DMI: Dry matter intake
DOMI: Digestible organic matter intake
EFE: Exogenous feed enzymes
ENU: Efficiency of nitrogen utilization
FAU: Fungal amylase unit
FBL: Xylanase treatment
FD: Full dose combination treatment
FNE: Feces nitrogen excretion
HD: Half dose combination treatment
IU: International unit
ME: Metabolizable energy
MN: Microbial nitrogen
N: Nitrogen
N_2_O: Nitrous oxide
NEg: Net energy for gain
NEm: Net energy for maintenance
NI: Not identifield
Nmic: Microbial nitrogen synthesis
N-NH_3_: Ruminal ammonia
OM: Organic matter
Pa: Purines absorbed
PDe: Purine derivative excreted
SEM: Standard error of the mean
TDN: Total digestible nutrients
TMR: Total mixed ration
UNE: Urine nitrogen excretion
VFA: Volatile fatty acids
