# Supplementary material for: Feeding amylolytic and fibrolytic exogenous enzymes in feedlot diets: effects on ruminal parameters, nitrogen balance and microbial diversity of Nellore cattle
Source: J Anim Sci Biotechnol. 2025 Jul 7;16:96. doi: 10.1186/s40104-025-01226-5 (PMC12232634; doi:10.1186/s40104-025-01226-5)
Supplement: Supplementary file 1 — Supplementary Material 1: Table S1 Total ASVs, richness and diversity index in cannulated Nellore finishing steers (n = 10) feeding EFE. Fig. S1 Principal Coordinate Analysis plot showing the variation between bacterial communities of cannulated Nellore finishing steers (n = 10) feeding diets with or without EFE. [file 40104_2025_1226_MOESM1_ESM.docx]

**Table S1** Total ASVs, richness and diversity index in cannulated Nellore finishing steers (*n* = 10) feeding EFE

| **Items^1^** |  | **Treatments^1^** | | | | |  | ***P*-value*^2^*** | | | |
| --- | --- | --- | --- | --- | --- | --- | --- | --- | --- | --- | --- |
|  |  | **CON** | **AML** | **FBL** | **HD** | **FD** |  | **C1** | **C2** | **C3** | **C4** |
| Total ASVs |  | 68,653±5046 | 66,936±9,795 | 68,786±10,084 | 69,948±9,601 | 70,570±7,708 |  | 0.796 | 0.508 | 0.959 | 0.721 |
| Bacteria |  | 97.3±2.46 | 97.5±1.86 | 97.9±2.10 | 97.5±2.13 | 97.5±2.12 |  | 0.916 | 0.386 | 0.799 | 0.646 |
| Archea |  | 2.70±2.46 | 2.49±1.85 | 2.13±2.10 | 2.51±2.13 | 2.53±2.13 |  | 0.917 | 0.386 | 0.799 | 0.646 |
| Richness |  |  |  |  |  |  |  |  |  |  |  |
| Chao 1 |  | 846±157 | 902±252 | 925±345 | 808±162 | 870±292 |  | 0.942 | 0.492 | 0.375 | 0.625 |
| Ace |  | 844±159 | 991±241 | 922±341 | 804±173 | 872±274 |  | 0.923 | 0.432 | 0.232 | 0.625 |
| Diversity |  |  | | | | | |  |  |  |  |
| Fisher |  | 156±35.8 | 166±55.4 | 178±78.9 | 145±36.7 | 162±606 |  | 0.913 | 0.375 | 0.193 | 0.695 |
| Simpson |  | 0.984±0.011 | 0.977±0.018 | 0.984±0.007 | 0.978±0.01 | 0.981±0.01 |  | 0.577 | 0.557 | 0.160 | 0.432 |
| Shannon-Wiener |  | 172±71.6 | 136±40.7 | 223±129 | 146±53.6 | 166±81.4 |  | 0.467 | 0.770 | 0.105 | 0.492 |

^1^Control (CON) = no feed additives; Amylase (AML) = amylolytic enzyme (Amaize, Alltech) added at 0.5 g/kg diet DM; Xylanase (FBL) = fibrolytic enzyme (Fibrozyme, Alltech) added at 0.9 g/kg diet DM; Half dose of Amylase and Xylanase (HD) = amylolytic enzyme added at 0.25 g/kg diet DM and fibrolytic enzyme (Fibrozyme, Alltech) added at 0.45 g/kg diet DM; and Full dose of Amylase and Xylanase (FD) = amylolytic enzyme added at 0.5 g/kg diet DM and fibrolytic enzyme added at 0.90 g/kg diet DM

^2^ (C1) CON vs. ENZ using the Kruskal-Wallis test. Paired Wilcoxon Rank Sum Test was applied to compare (C2) AML vs. FD, (C3) FBL vs. FD, and (C4) HD vs. FD

ASVs = amplicon sequence variants

**
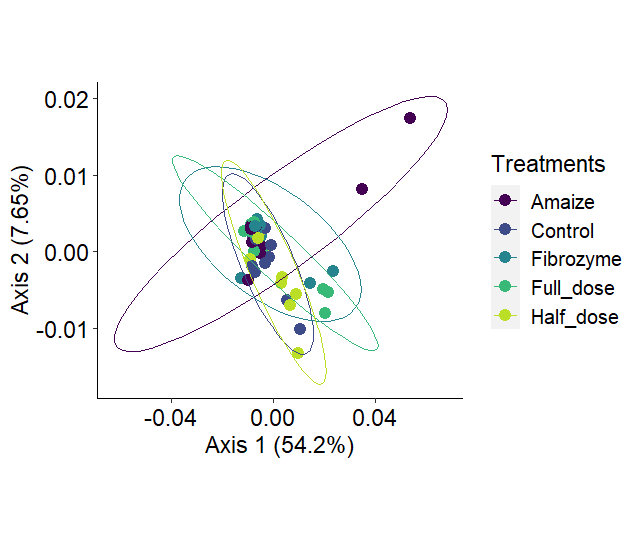
**

**Fig. S1** Principal Coordinate Analysis plot showing the variation between bacterial communities of cannulated Nellore finishing steers (*n* = 10) feeding diets with or without EFE
